# The use of probiotics in animal feeding for safe production and as potential alternatives to antibiotics

**DOI:** 10.14202/vetworld.2021.319-328

**Published:** 2021-02-03

**Authors:** Mbarga M. J. Arsène, Anyutoulou K. L. Davares, Smolyakova L. Andreevna, Ermolaev A. Vladimirovich, Bassa Z. Carime, Razan Marouf, Ibrahim Khelifi

**Affiliations:** 1 Department of microbiology and virology, Institute of Medicine, RUDN University, Moscow, Russia; 2 Department of Food Sciences and Nutrition, National School of Agro-industrial Sciences, University of Ngaoundere, Cameroon; 3 Department of Process Engineering, National School of Agro-industrial Sciences, University of Ngaoundere, Cameroon

**Keywords:** animal nutrition, antibiotic resistance, feed additive, fish farming, pig poultry, probiotic, safe production

## Abstract

Although the production of safe food for human consumption is the primary purpose for animal rearing, the environment and well-being of the animals must also be taken into consideration. Based on microbiological point of view, the production of healthy food from animals involves considering foodborne pathogens, on the one hand and on the other hand, the methods used to fight against germs during breeding. The conventional method to control or prevent bacterial infections in farming is the use antibiotics. However, the banning of these compounds as growth promoters caused many changes in animal breeding and their use has since been limited to the treatment and prevention of bacterial infections. In this function, their importance no longer needs to be demonstrated, but unfortunately, their excessive and abusive use have led to a double problem which can have harmful consequences on consumer health: Resistance to antibiotics and the presence of antibiotic residues in food. The use of probiotics appears to be a suitable alternative to overcome these problems because of their ability to modulate the immune system and intestinal microflora, and further considering their antagonistic role against certain pathogenic bacteria and their ability to play the role of growth factor (sometimes associated with prebiotics) when used as feed additives. This review aims to highlight some of the negative effects of the use of antibiotics in animal rearing as well as emphasize the current knowledge on the use of probiotics as a feed additive, their influence on animal production and their potential utility as an alternative to conventional antibiotics, particularly in poultry, pig, and fish farming.

## Introduction

In most developed countries, the amount of protein consumed is on average above 1.4 g/kg daily and animal proteins represent 65-70% of the total protein intake [1]. To meet the growing demand, livestock industries of all classes have resorted to various techniques to increase their output [2]. In addition to setting up intensive and semi-intensive farming system, these new techniques are particularly intended to accelerate the growth of animals, protect them against diseases, improve feed conversion efficiency, and optimize reproduction by limiting the mortality rates during birth [3].

Antibiotics have long been used in animal husbandry as growth promoters, on the one hand and on the other hand, as treatment for bacterial diseases [4]. The prohibition of antibiotics as growth promoters and the harmful effects resulting from its abusive use (resistance to antibiotics and presence of antibiotic residues in food and the environment) increasingly force breeders to look for other more eco-efficient methods [5]. From the beginning of 21^st^ century, several studies have been carried out presenting probiotics as being able to play a preponderant role in breeding, either as a simple additive with beneficial effects on growth or as a potential alternative to conventional antibiotics [6-8]. Numerous recent studies have shown that supplementing probiotics in animal feed positively alter the gut microbiota, reduce pathogen shedding and disease symptoms, increases gut immunity, and improve disease resistance and health [9-15]. In addition, probiotics have their antagonistic effect and their ability to regulate the gut microflora can significantly reduce foodborne pathogens such as Campylobacter, *Clostridium perfringens*, *Escherichia coli*, *Listeria monocytogenes*, Salmonella, and *Staphylococcus aureus* [16-19].

This review, therefore, serves to highlight the applications of probiotics in animal breeding (pig, poultry, and fish) as well as their potential utility as an alternative to conventional antibiotics.

## Harmful Consequences of the Use of Antibiotics in Animal Breeding

### Resistance to antibiotics

**Figure-1:**
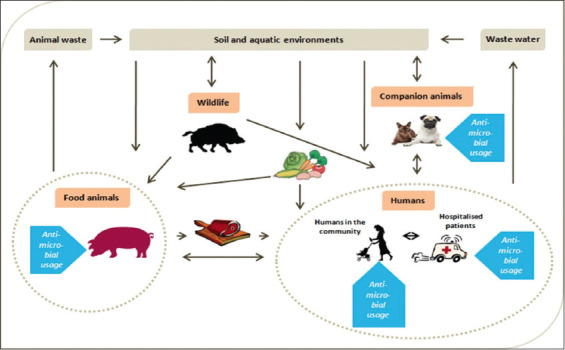
Exchange of resistance genes and bacteria between different reservoirs [5].

Antibiotic resistance is defined as the ability of bacteria to resist the inhibitory or destructive activity of an antibiotic to which it was not resistant [20]. This resistance, in particular, is associated with excessive or sometimes uncontrolled use of antibiotics in breeding and the mechanisms of transmission of resistance between bacteria. This phenomenon concerns both animals and humans and the transmission of resistance between them is possible because they share the same ecosystem (Figure-1). Indeed, resistant bacteria, resistance genes (especially associated with mobile genetic elements), and antibiotic residues can circulate between the different niches of the ecosystem [5]. By direct or indirect contact (food, water, and environment) between animals and human, bacteria can, therefore, pass from animals to human, and on the contrary. This applies both to commensal bacteria, which are often considered as reservoirs of resistance due to their widespread presence and long-term antibiotic exposure in the gut of food animals (use of antimicrobial growth promoters at subtherapeutic levels), and also to pathogenic bacteria [5]. Furthermore, recent studies have shown that multi-resistant bacteria from animal breeding can be found in everyday consumer products [21]. Resistance to antibiotics can be very negative for the production of meat, milk, and other animal products if the prevention and treatment of bacterial infections with antibiotics becomes completely obsolete due to antibiotic resistance. Likewise, bacteria that have acquired resistance to several antibiotics could be twice as dangerous if transmission to humans is effective and especially if they are pathogenic, because the treatment of the disease induced would be more difficult [4].

### Residues of antibiotics in food and environment

One of the major consequences of the use of antibiotics in agriculture and animal husbandry is the presence of residues of these substances in food and environment. Antibiotic residues are defined as all pharmacologically active substances, whether active substances, excipients, or degradation products, as well as the metabolites found in the environment or remaining in food for animals to which the drug in question has been administered [22]. These residues may pose risks to public health, in particular with the development of resistance (due to exposure of bacteria to underdoses), allergic reactions, disruption of normal flora, and potential carcinogenicity, mutagenicity, and teratogenicity [4]. With regard to the environmental risks, it is presently accepted that after an antibiotic treatment, the animals excrete in their environment a fraction of the administered dose [5]. Most certainly, there are significant disparities in the half-life time depending on the molecule: Tylosin, for example, degrades much faster than oxytetracycline, detectable in the manure of calves treated for 5 months against <45 days for tylosin. This signifies a long persistence of certain antibiotics in the environment, which can then be present in soil, water, and rivers, therefore leading to chemical pollution of the environment, with an action on the microbial flora which may be the same as on the commensal flora [23]. Similarly, technological risks should not be overlooked because antibiotic residues can interfere with the fermentation process during the production of certain fermented foods (cheese, yogurt, and fermented meat) by inhibiting the starter cultures [4].

## Use of Probiotic in Animal Breeding

Probiotics are live microorganisms that confer health benefits on the host when administered in adequate dosage [24]. Their use in human health and farm animal production has been widely reported in the literature. Although several microorganisms, particularly bacteria and fungi, have demonstrated probiotic abilities, species belonging to the genera *Lactobacillus*, *Streptococcus*, *Lactococcus*, and Bifidobacterium remain the most popular probiotic agents to date [25]. Very often, probiotic candidates must at least be able to modulate the immune system or certain physiological parameters of the host, attenuate virulence markers of certain pathogens, treat or prevent infectious, and inflammatory diseases and act as a biological control agent in the prevention of spoilage [26]. There are reports indicating that the use of probiotic yeast (*Saccharomyces cerevisiae*) and fungal strains (*Aspergillus oryzae*) provides better results in adult ruminants while bacterial probiotics are more effective in chickens, pigs, and young calves [26].

### Most used probiotics bacteria and application in animal breeding

#### Lactobacillus

*Lactobacillus* is Gram-positive bacteria belonging to the group of lactic acid-producing bacteria. This group is very wide and heterogeneous and includes more than 100 different species [27]. Most of the species found in this group are part of the normal mammal’s microbiota. Many species belonging to this genus are very often introduced as probiotics in both dairy and non-dairy foods intended for human consumption [24,28]. Meanwhile, some species of the genus *Lactobacillus* used as feed additive have demonstrated beneficial abilities, in particular, to reduce mortality in fish [6,29], to improve growth performance in piglets [30], to improve production and quality of eggs in poultry [7,16,31], to improve immune defense mechanisms in fish [32], and to reduce Salmonella contamination in chicken [5-7,9-19,25,29-78] (Table-1). Otherwise, *Lactobacillus* strains producing active dietary enzymes including protease amylase, lipase, phytase, and protease are interesting probiotic candidates due to these enzymes’ role in digestion and absorption of nutrients [79]. However, Vesterlund *et al*. [80] mentioned that some bacteria of this genus (such as *Lactobacillus casei* and *Lactobacillus rhamnosus*) could be involved in bacterial infections. Notwithstanding the above, most authors agree that this large group is recognized as generally safe for both animals and humans.

**Table-1:** Overview of some probiotics used in animal feeding: The properties and its use as alternative to antibiotics.

Genus	Species	Type of breeding	Effect	Sources
*Lactobacillus*	*L. fermentum*^ab^	Piglets^ab^	• Increase growth performances • Decrease *E. coli* counts and reduce post-weaning diarrhea	[30]
		Grower-finisher pigs^a^	• Enhance superoxide dismutase, glutathione peroxidase, and catalase	[50]
		Poultry^ab^	• Regulate the intestinal mucosal immune response and ameliorate inflammation caused by *Clostridium perfringens*	[9]
	*L. sobrius*^ab^	Piglets^ab^	• limit intestinal colonization by pathogenic *E. coli.*	[51]
	*L. salivarius*^ab^	Swine^ab^ and poultry^ab^	• Improve the immune status and reduce colonization by pathogenic bacteria in swine and poultry	[10]
	*L. rhamnosus*^ab^	Fish^ab^	• Reduce mortality in Rainbow trout fish affected by *A. salmonocida* and in Tilapia affected by *Edwardsiella tarda*	[6,29]
	*L. reuteri^a^*	Piglets^a^	• Beneficial effects on the expression of tight Junction proteins in newborn piglets (L. *reuteri* I5007)	[11]
	*L. plantarum*^ab^	Fish^ab^	• Reduce mortality in Rainbow trout fish affected by *Lc. garvieae*	[12]
		Grower-finisher pigs^a^	• Enhance superoxide dismutase, glutathione peroxidase and catalase	[52]
	*L. acidophilus*	Chicken^ab^	• Increase eggs production and improve eggs quality • Reduce Salmonella contamination	[7,16,31]
		Piglets^ab^	• Increase growth performances; • Decrease *E. coli* counts and reduce post weaning diarrhea	[30]
	*L. amylovorus*^ab^	Pigs^ab^ and piglets^ab^	• Inhibit diarrheal pathogens in pigs • Protect against enterotoxigenic *E. coli* (ETEC) infection by cytokine regulation in piglets	[53,54]
	*L. paracasei*^ab^	Piglets^ab^	• limit intestinal colonization by pathogenic *E. coli*	[55]
	*L. pentosus*^ab^	Fish^ab^	• Improve immune system and survival rate of Japanesse eel fish affected by *Edwardsiella tarda*	[56]
	*L. brevis*^ab^	Fish^ab^	• Reduce mortality of Tilapia affected by *A. hydrophilia*	[57]
	*L. casei*^ab^	Piglets^ab^	• Increase growth performances; • Decrease *E. coli* counts and reduce post-weaning diarrhea	[30]
	*Lc. lactis*^ab^	Fish^ab^	• Activate the innate immune system of Olive flounder fish and protect against pathogen infections	[32]
Bifidobacterium	*B. animalis*^ab^	Poultry^ab^	• Reduce coccidiosis in broiler chickens infected with *Eimeria tenella*	[17,18]
	*B. thermophilum*^ab^	Poultry^ab^	• Have protective activity against Salmonella and Listeria spec. *in vitro* and against *E. coli* in chicken	[34]
	*B. longum*^ab^	Poultry^ab^	• Have an anti-Campylobacter activity in poultry	[35]
	*B. pseudolongum*^a^	Piglets^a^	• Better FCR with no differences in final weight, weight gain and feed intake	[33]
Bacillus	*B. licheniformis*^ab^	Pig^ab^	• Effective against diarrhea occurring in piglets in 3–10 days post-weaning caused by enterotoxic strains of *E. coli*	[19]
	*B. subtilis*^ab^	Fish^ab^	• Control of infection in Indian major carp • Increase survival rate of rainbow trout • Reduce mortality of channel catfish and striped catfish • Enhance the relative survival percentage of grouper fish	[36,39,58]
		Broilers^ab^	• Improve productivity and reduces Salmonella in broilers • Reduce the *Salmonella gallinarum* pathogens	[37,38]
	*B. pumilus*^ab^	Fish^ab^	• Enhance the immune and health status and improve disease resistance of Tilapia	[59]
	*B. circulans*^ab^	Fish^ab^	• Enhance the immune response and therefore survival of Catla catla fish	[60]
*Enterococcus*	*E. faecalis*^ab^	Fish^ab^	• protective effect against *Lc. garvieae* and potential alternative for controlling diseases in aquaculture	[61]
	*E. faecium*^ab^	Tuckey^a^	• Stimulates other lactic acid bacteria in the small intestine, especially lactobacilli	[40]
		Chicken^ab^	• Significantly improved weight gain and food conversion rate (FCR) • Efficient in controlling and reducing the counts of *Salmonella minnesota*	[41,62]
		Broilers^ab^	• Promote growth performance • Improve intestinal morphology, and beneficially manipulate the cecal microflora	[63]
		Fish^ab^	• Reduce Edwardsiellosis in European eel fishes	[64]
		Piglets^ab^	• Significantly modulate the fecal microbiome of weaned pigs • bacterial diversity and increase beneficial bacteria and decrease pathogenic bacteria • Increase the average of daily gain	[65]
	*E. gallinarum*^ab^	Fish^ab^	• Have moderate protective effect on Seas bass fishes	[13]
	*E. casseliflavus*^ab^	Fish^ab^	• Improve growth performance and enhances disease resistance due to *S. iniae* on Rainbow trout fishes	[14]
*Lactococcus*	*Lc. lactis*	Fish^ab^	During *Aeromonas salmonicida* infection in Brown Trout (*Salmo trutta*) • Improves survival rate • Activates phagocytic cells in the head kidney • Reduces the rate of proliferation of pathogens in the intestine	[42]
		Swine^b^	• Useful as an effective subunit vaccine against swine erysipelas	[66]
*Leuconostoc*	*L. mesenteroides*	Fish^ab^	• Improves survival rate, activates phagocytic cells in the head kidney, reduces the rate of proliferation of pathogens in the intestine during *A. salmonicida* infection in in Brown Trout	[42]
		Poultry^ab^	• *L. mesenteroides* isomaltooligosaccharides stimulate growth of Bifidobacterium and *Lactobacillus* and are not used by Salmonella or *E. coli*	[67]
*Pediococcus*	*P. acidilactici*^a^	Chickens^a^	• Improved the feed conversion ratio of broiler chickens	[15]
*Streptococcus*	*S. thermophilus*^a^	Chickens^a^	• Improved the feed conversion ratio of broiler chickens	[15]
	*S. phocae*^ab^	Fish^ab^	• significantly improve growth performance as well as protection against *V. harveyi* in *P. monodon* post-larvae	[68]
Aspergillus	*A. orizae*	Chickens^ab^	• Enhance body weight gain and feed intake	[69]
	*A. niger*^a^	Broilers^a^	• Improved growth performance; • Improve the growth performance, decrease the muscle protein breakdown, abdominal fat content and cholesterol content in plasma	[70,71]
Saccharomyces	*S. cerevisiae*^ab^	Sows and piglets^ab^	• Live yeasts improve reproductive performance of sows • Elevate IgG concentration in colostrum and subsequently plasma IgG of piglets • Improve growth performance • Promote a “healthy” intestine • Encourage an early restoration of the intestinal mucosal	[43-45,72]
		Fish^ab^	• Improve growth, hematological, antioxidant, and immune responses of Nile tilapia • Improve resistance of Nile tilapia against pathogenic fungus *A. flavus* infection • Enhances the cellular innate immune response of gilthead seabream (*Sparus aurata* L.)	[5,25,46,47]
Kluyveromyces	*K. fragilis*^ab^	Piglets^ab^	• Improve innate immune system parameters such as phagocytic activity of monocytes and improve fecal scores.	[73]
	*K. marxianus*^ab^	Fish	• Protein sources in diets for Atlantic salmon when taken as spray-dried yeasts	[74]
		Broilers^ab^	• Contributes to food efficiency; • Improves immune function and intestinal structure of broilers; • The high doses (2.5 g/kg) are effective for feed efficiency and intestinal health of chickens • Average doses (1.0 g/kg) optimize innate immunity (1.0 g/kg).	[75]
Combinations	BioPlus 2B: *B. licheniformis and B. subtilis spores*^ab^	Sow and piglets^ab^	• Improve the health status and fertility of sows • Increase of sow feed consumption during the first 14 days postpartum • Decrease in piglet diarrhea score • Increase in the number of weaned piglets per litter • Increase in piglet body weight at weaning. • Decrease of sow weight loss during the suckling period. • Decrease in preweaning mortality	[48]
	Dietary probiotics *Lc. lactis BFE920 and Lactobacillus plantarum FGL0001*	Fish^ab^	• The mixture-fed significantly improved innate immunity and weight gain of olive flounder (*Paralichthys olivaceus*). • High survival against *S. iniae* infection in olive founder	[76]
	Dietary *Lactobacillus reuteri, L. salivarius and Streptococcus salivarius*^ab^	Piglets^ab^	• Significantly improved the growth performance, blood parameters and IgG stimulation in weaned piglets	[77]
	LACTINA^®^: *L. acidophilus, L. helveticus, L. bulgaricus, Lc. lactis, S. thermophiles* and *E. faecium*	Piglets^ab^	• Increase the body weight of suckling piglets when used at approximately 5 × 10^9^ CFU/kg feed.	[49]
	Dietary supplementation of a mixture of *Lactobacillus pentosus* ITA23 and *Lactobacillus acidophilus* ITA44	Chickens	• Positively affects the final body weight under low (24°C) and high (35°C) temperature conditions; • Improves the average daily gain (ADG); • Increases the expression of the four sugar transporter genes: GLUT2, GLUT5, SGLT1, and SGLT4; • Improves bacterial population of the cecal contents, by increasing beneficial bacteria and decreasing *E. coli* population	[78]

a=Used at least once as feed additive, b=Used at least once as alternative to antibiotics. *E. coli: Escherichia coli, S. cerevisiae: Saccharomyces cerevisiae, L. paracasei: Lactobacillus paracasei, L. casei: Lactobacillus casei, L. rhamnosus: Lactobacillus rhamnosus, Bifidobacterium pseudolongum: Bifidobacterium pseudolongum, B. animalis: Bifidobacterium animalis, B. thermophilum: Bifidobacterium thermophilum, B. longum: Bifidobacterium longum, E. tenella: Eimeria tenella, B. subtilis: Bacillus subtilis, B. licheniformis: Bacillus licheniformis, E. faecium: Enterococcus faecium, S. iniae: Streptococcus iniae, Lc. lactis: Lactococcus lactis, Lc. garvieae: Lactococcus garvieae*, IgG: Immunoglobulin G

#### Bifidobacterium

Bifidobacteria are found in large numbers in the gut of animals and human. It is a very promising group as a probiotic and its presence in the gut generally indicates the good health of the host [27]. There is a general belief that this bacterium has a significant contribution in maintaining the balance of the intestinal microflora and in limiting the risk of infections. Several species are host specific [8]. Many bifidobacteria are generally used as probiotics in human food and in pharmaceutical formulations [27]. Many of these germs have “GRAS” (“Generally Regarded As Safe”) status [33]. Used as a feed additive in piglets, the species *Bifidobacterium pseudolongum* have shown significant results on a better food conversion ratio (FCR) with no differences in final weight, weight gain, and feed intake [33]. In poultry, the species *Bifidobacterium animalis*, *Bifidobacterium thermophilum*, and *Bifidobacterium longum*, used as food additive have, respectively, demonstrated their ability to reduce coccidiosis in broiler chickens infected with *Eimeria tenella* [17,18], to have protective activity against Salmonella and Listeria species *in vitro* and against *E. coli* in chicken [34], and an anti-Campylobacter activity [35]. Overall, bacteria belonging to the Bifidobacterium genus are extensively tested for their potential application as a feed additive and as an alternative to conventional antibiotics in breeding. The results obtained are promising and their ability to specifically inhibit certain pathogens is a major asset.

#### Bacillus

Bacillus are Gram-positive bacteria, facultative aerobic or aero-anaerobic, heterotrophic, saprophytic, and ubiquitous. Some bacteria of this genus such as *Bacillus subtilis* are regularly used as a food supplement in animal breeding, especially in fish farming [36] and in poultry [37,38]. Kumar *et al*. [81] reported that feeding the Indian big carp *Labeo rohita* with *B. subtilis* at 1.5 × 10^7^ CFU/g contributed to increased resistance against infection with *A. hydrophila. B. subtilis*, administrated at 10^4^, 10^6^, and 10^8^ CFU/g for 14 and 28 days, has also demonstrated its ability to enhance the relative survival percentage of groupers, *Epinephelus coioides* challenged with *Streptococcus* spp. [39]. Several researchers agree that these species possess high potential for immunomodulation and protection against diseases in animal breeding, and recommend *B. subtilis* as a beneficial agent for the biological control of the diseases [25]. Otherwise, other species of the genus Bacillus like *Bacillus licheniformis* have also shown probiotic aptitudes, when used as a feed additive in pigs, and have shown to be effective against diarrhea occurring in piglets in 3-10 days post weaning caused by enterotoxic strains of *E. coli* [19]. Although some species like *Bacillus cereus* can cause problems due to the endotoxins and emetic toxins they produce [82], bacteria of the genus Bacillus already used as probiotics have real potential and can be used in safe production and as an alternative to conventional antibiotics.

#### Enterococcus

*Enterococcus* is a common member of the endogenous intestinal microbiota of humans and animals [83]. Although this genus is not considered “generally recognized as safe”, species from the genus Enterococcus have been used as probiotic for human or animals [83,84]. *Enterococcus* strains have been used as feed additives in poultry and swine as alternatives to the use of sub-lethal antibiotics in the feeds. Several studies aimed to evaluate the probiotic aptitudes of species of the genus *Enterococcus* have been carried out, and most of these studies focused on *Enterococcus faecium*. In a study conducted by Pollmann *et al*. [85] to investigate the impact of probiotic additives on the rate of endogenous Chlamydia infection in pigs, a microencapsulated *E. faecium* SF68 (NCIMB 10415), containing 9×10^9^ CF/g bacteria has shown positive results and a reduction in the severity of infections as well as the number of infections caused by Chlamydiae. The strain *E. faecium* has also demonstrated its ability to stimulate other lactic acid bacteria (especially *Lactobacillus*) in the small intestine of a turkey [40], improve the FCR in chicken [41], improve intestinal morphology, and beneficially manipulate the cecal microflora in broilers [9] (Table-1). Some other beneficial effects of *E. faecium* and other strains of the *Enterococcus* genus on poultry, pigs, and fish are listed in Table-1. However, the *Enterococcus* genus does not only have advantages, as these bacteria may participate in transmission of resistance to antibiotics [82]. Moreover, this genus is often associated with pathogenesis such as infections of the urogenital tract [86] and endocarditis [87]. Sometimes, these strains are involved in the production of substances such as β-hemolysin, gelatinase, and aggregation substance that are undesirable phenotypes in probiotic strains [83]. Despite having demonstrated good results in animal breeding, the use of probiotics belonging to this genus must be checked beforehand to use only strains which do not present any danger to the health of the animal.

#### Lactococcus

*Lactococcus* strains are commonly used in the manufacture of fermented dairy products. Some of them have been tested for probiotic properties, especially in fish and the results have proven to be satisfactory and promising [42]. Special interest is placed on the study of *Lactococcus lactis* and this species was capable of protecting different fish species against bacterial pathogens [25]. In a study conducted by Balcázar *et al*. [42], *Lc. lactis* administered to brown trout as a feed additive revealed its ability to increase immune parameters as well as protect against furunculosis. In the same vein, Heo *et al*. [88] reported the use of *Lc. Lactis* (10^8^ CFU/g) in olive flounder elevated serum immune parameters (such as serum peroxidase, lysozyme, antiprotease, and blood respiratory burst activities) as well as resistance against *Streptococcus iniae*. Although *Lc. lactis* is considered safe for human and animal use, some studies have also linked *Lactococcus* bacteria (*Lc. lactis* and *Lactococcus garvieae*) to infection [89]. It is, therefore, necessary to regularly carry out trials before implementing the strains of this genus in animal feed, either as an additive or as a means of disease control.

#### Saccharomyces

Saccharomyces is a genus of budding yeast; it is also part of the gut microbiota. In this fungal genus, *S. cerevisiae* is the best-known specie and most used as a probiotic. *S. cerevisiae* has been recognized to improve reproductive performance of sows elevate immunoglobulin G (IgG) concentration in colostrum and subsequently plasma IgG of piglets, to improve growth performance and promote “healthy” intestines of pigs [43-45]. Positive results of the use of *S. cerevisiae* have also been observed in fish. *S. cerevisiae* has been shown to be able to improve growth, hematological, antioxidant, and immune responses of Nile tilapia, improve resistance of Nile tilapia against pathogenic fungus *A. flavus* infection as well as enhance the cellular innate immune response of gilthead seabream [25,46,47,90]. Other species belonging to this genus such as *Saccharomyces carlsbergensis* are also used as probiotics in animal feeding [27].

#### Other probiotics and combinations of probiotics

Certain other strains used as probiotics in animal feed as well as their positive effect on the host are given in Table-1. Furthermore, although the benefits of using a combination of more than one probiotic in the same food formulation are not yet demonstrated, most commercial products such as BioPlus 2B [48] and LACTINA^®^ [49] contain various probiotic strains (Table-1).

## Conclusion

The beneficial properties of probiotics when used as a feed additive are very encouraging for animal breeding. The study of the existing literature on this subject allowed us to highlight their potential use as an alternative for conventional antibiotics. However, more research should be done to standardize the use of specific probiotic strains in the breeding of specific animals, while maintaining the properties already demonstrated.

## Authors’ Contributions

MMJA, AKLD, and RM conceptualized and designed review, literature search, and wrote the first manuscript draft. SLA, EAV, BZC, and IK edited and revised the final draft of the review article. All authors critically reviewed the manuscript and gave final approval of the version to be published.
